# Genotyping *KIF1C* (c.608G>A) Mutant Reveals a Wide Distribution of Progressive Ataxia in German Charolais Cattle

**DOI:** 10.3390/ani14030366

**Published:** 2024-01-23

**Authors:** Felix Manuel Bischofberger, Sina Reinartz, Ottmar Distl

**Affiliations:** Institute of Animal Genomics, University of Veterinary Medicine Hannover (Foundation), 30559 Hannover, Germany; felix.bischofberger@tiho-hannover.de (F.M.B.); sina.reinartz@amedes-group.com (S.R.)

**Keywords:** daily weight gain, muscle conformation, estimated breeding value, additive and dominant effect, pleiotropy, linkage disequilibrium

## Abstract

**Simple Summary:**

Progressive ataxia is an autosomal recessive defect that is lethal due to the irreversible changes it causes to the brain and spinal cord. Only homozygous carriers of the mutated *KIF1C* allele manifest progressing clinical signs, mostly no earlier than 12–24 months of age. The objectives of this field study were to determine the prevalence of the mutated *KIF1C* allele, uncover its associations with growth and muscle conformation, and build awareness of this lethal condition among beef cattle breeders in Germany. A total of 1315 samples was sent in for mutation testing. Prevalence of the mutated *KIF1C* allele was 11.75%, resulting from 293 heterozygous and 8 homozygous mutant animals. Homozygous carriers of the mutated *KIF1C* allele showed a significant superiority in estimated breeding values for daily weight gain and muscle conformation. In order to reduce the frequency of the mutated *KIF1C* allele, genetic testing should be introduced for Charolais cattle and should be mandatory for all Charolais breeding bulls. It is important to create awareness of this condition among beef cattle breeders, in addition to the possibilities of preventing the number of affected animals. In addition, genetic testing of eight further beef, dual-purpose, or dairy cattle breeds revealed that two German Angus cattle showed heterozygous mutations for the *KIF1C* A-allele; therefore, we recommend genetic testing of German Angus to prevent the spread of the *KIF1C* allele.

**Abstract:**

Bovine progressive ataxia in Charolais cattle was first described in the 1970s; then, cases were reported in Charolais worldwide. A homozygous loss-of-function mutation within the *KIF1C* gene (c.608G>A) was found to be responsible for this neurodegenerative disease. The aim of this study was to determine whether the mutated *KIF1C* allele segregates in the German Charolais population and whether the estimated breeding values for growth and muscle conformation are associated with the mutated genotypes. Genetic test results of the *KIF1C:c.608G>A* variant were available for 1315 Charolais cattle from 35 herds located in Germany. In addition, 324 samples from eight other beef cattle breeds were tested for the mutated *KIF1C* allele. We were able to demonstrate that the *KIF1C* mutation is common, with a frequency of 11.75% in the German Charolais population. All but two of the eight (2/8 = 25%) homozygous mutated individuals showed clinical signs consistent with progressive ataxia. The estimated breeding values of muscle conformation in 200- and 365-day-old animals indicated a significant superiority for homozygous mutated animals when compared either with heterozygous or homozygous wild-type genotypes; this was also the case for heterozygous genotypes in comparison with homozygous wild-type genotypes. For the estimated breeding values of daily weight gain in 200- and 365-day-old animals, the significant differences between homozygous mutated and heterozygous or wild-type genotypes were in favour of the homozygous mutant animals. There were no differences in the estimated maternal breeding values among all three *KIF1C* genotypes. For the first time, two German Angus cattle carrying the *KIF1C* mutation heterozygous were detected. The breeders’ survey highlighted that increased awareness would facilitate increased conviction among breeders of the need for genetic testing in order to eliminate the lethal *KIF1C* allele.

## 1. Introduction

Progressive ataxia is an inherited neurodegenerative disease that is specific to Charolais and any Charolais crossbreeds [1,2,3,4,5,6,7,8,9,10]. The first cases were seen in the early 1970s in England [1,2,3]. Further cases in this breed were reported in New Zealand [4], the United States [5,6,7], Australia [8], and Scotland [11]. An increasing number of affected Charolais cattle has been found in France since the 2000s; thus, in order to prevent the further dissemination of this lethal disease, an efficient, molecular–genetically based breeding program is urgently needed [10]. Homozygosity mapping and whole-genome sequencing of affected Charolais cattle showed that 60/70 cases were concordant with a homozygous loss-of-function mutation within the *kinesin family member 1* (*KIF1C*) gene (gene ID: 514928, c.608G>A, p.R203Q) [10]. The mutant *KIF1C* allele was estimated at 13% in French Charolais cattle. Screening Limousin, Brown Swiss, Montbeliard, Holstein, and Blonde d’Aquitaine detected the presence of the mutated *KIF1C* allele in Blonde d’Aquitaine with Charolais ancestry only [10]. Similarly, in the Uckermärker breed, which was created through crossbreeding German Fleckvieh cows with Charolais bulls, the presence of the *KIF1C* mutation was shown with a frequency of 12% [9]. Introgression of the mutated *KIF1C* allele came about through Charolais bulls before 1985 and has persisted in the Uckermärker population ever since. These reports highlight the dissemination of the lethal *KIF1C* mutation into cattle populations crossbred with Charolais [6,8,10]. *KIF1C* mutations in humans are responsible for spastic ataxia or complex hereditary spastic paraplegia [12,13,14]. Clinical signs in homozygous mutated Charolais cattle become evident at 12–24 months of age; in rare cases, signs show as early as six months or as late as five years of age [1,5,8,10]. Ataxia mostly involves the hind limbs. Involvement of all four limbs has only been recorded in some cases. Limb incoordination progresses within a few months after early clinical signs like slight incoordination. Further progression leads to difficulty in rising and permanent recumbency. Other signs are irregular urine heel, and head tremor when excited [1]. An effective treatment has not been described [1,2,3].

Heterozygous carriers of the deleterious *KIF1C* allele exhibit an advantage in growth rate and muscular development compared to wild-type homozygous Charolais cattle [10]. This may explain the high frequency of the mutated *KIF1C* allele in French Charolais cattle and possibly in Uckermärker cattle [9,10]. 

The prevalence of the mutated *KIF1C* allele has not yet been reported in Charolais populations other than in the French population. Therefore, reports from other Charolais populations should show whether the mutated *KIF1C* allele frequency is different in comparison to the French population and whether a selective advantage of the mutated *KIF1C* allele may be confirmed. The aims of our study were as follows: to investigate randomly sampled German Charolais cattle for the mutated *KIF1C* allele; to compare the *KIF1C* genotypes with the phenotypic abnormalities of the animals reported by the farmers, particularly when animals had a history of incoordination and ataxic movements; to compare growth traits with the *KIF1C* genotypes in breeding animals. In addition, we performed a survey among farmers on the awareness of progressive ataxia in Charolais cattle and how they take care to prevent dissemination of the mutated *KIF1C* allele. The importance of this study may be underlined by the fact that the Charolais breed, with over 120,000 individuals, is the most-used beef cattle breed in Germany after Limousin [15].

## 2. Materials and Methods

### 2.1. Ethical Approval

The study was performed according to the guidelines of the Declaration of Helsinki and approved by the Institutional Review Board of the University of Veterinary Medicine Hannover (Foundation) and the responsible state veterinary office of Lower Saxony (registration number 33.9-42502-05-18A253) on 25 January 2018. The sampling and handling of the animals followed European Union guidelines for animal care and handling and the Guidelines of Good Veterinary Practices.

### 2.2. Sample Collection

A submission form was made available for Charolais beef farmers and veterinary practitioners on our homepage (https://www.tiho-hannover.de/kliniken-institute/institute/institut-fuer-tierzucht-und-vererbungsforschung/forschung/forschungsprojekte-rind/progressive-ataxie (accessed on 18 September 2023)); additionally, beef cattle farmers were informed by their local breeding organizations on the availability of the genetic test for the *KIF1C* mutation (Supplementary Table S1). All participating farmers agreed that the genotyping results could be used for scientific purposes. Hair root samples were collected by farmers according to detailed instructions. We provided resealable laboratory plastic bags for each individual animal. For each animal, a different plastic bag had to be used; these were identified using a running number, the life number, the birth date, the sex, the sire, and the farm’s identification. In addition, a detailed sample submission form had to be filled in for each animal. Cross-contamination with hair roots of other animals had to be avoided. EDTA blood samples were collected by veterinarians. Sample collection was restricted to Monday–Wednesday to ensure the arrival of samples at our lab before Saturday.

In total, samples of 1315 Charolais cattle from 35 farms were included in this study. The animals examined contained 1109 female and 206 male individuals. The samples studied were obtained between May 2019 and June 2021. Sampling per herd was random but breeders were encouraged to include as many of their Charolais cattle as possible. In addition, we tested 324 samples from healthy animals as controls, including eight different cattle breeds.

### 2.3. DNA Extraction and Genotyping

Genomic DNA was extracted from the EDTA-blood samples and hair roots using routine procedures. Concentration of extracted DNA was determined using the Nanodrop ND-1000 (Peqlab Biotechnology, Erlangen, Germany) and adjusted to 10 ng/µL. After DNA extraction and concentration adjustment, genotyping of the *KIF1C*:c.608G>A variant was performed through a PCR-based restriction fragment length polymorphism (RFLP) test using the restriction enzyme BstUI and primer pairs according to Duchesne et al. [10] (Supplementary Materials Table S2 and Figure S1).

### 2.4. Pedigree Data and Breeding Value Estimation

In coordination with the German Charolais breeding association, we obtained access to pedigree data and estimated breeding values (EBVs) for all the examined animals which were registered in the herdbook. The data were provided by Vereinigte Informationssysteme Tierhaltung w.V. (vit), Verden/Aller, Germany.

The 200-day weights (weaning weight, WW 200d) and muscling scores (MC 200d) as well as the 365-day weights (yearling weight, YW 365d) and muscling scores (MC 365d) for all male and female breeding animals are recorded in the herdbook’s data. Herd testing involves all young animals between 90 and 500 days of age in at least two annual weighing cycles. Recordings are carried out according to the guidelines of the International Committee for performance testing in animal production (ICAR). The weights are recorded by employees of the state control association or by the owners. Muscular development scores have to be recorded by employees of the state control association. Muscling scores are determined according to the uniform regulations and the evaluation system of the Federal Association for Cattle and Pig (Bundesverband Rind und Schwein e.V., BRS, Bonn, Germany). Muscling is evaluated for the haunch, back, pelvic area, shoulder, and breast using a grading system on a 9-point scale. The final score for MC is summarized over these body parts by giving haunch and back three times higher weights (3/9) than the other body parts (1/9). Final scores are between 1 (very poor) and 9 (excellent) [16,17].

EBVs based on a best linear unbiased prediction (BLUP) multi-trait animal model are routinely calculated through vit/Verden for daily weight gain at day 200 (EBV-DG 200d), muscle conformation at day 200 (EBV-MC 200d), daily weight gain at day 365 (EBV-DG 365d), and muscle conformation at day 365 (EBV-MC 365d). In addition, maternal EBVs are provided for daily weight gain at day 200 (EBV-mat-DG 200d).

The comparison group is defined within a herd and a calving year (1.12.–30.11.). In order to reduce the proportion of herd-years with samples of just a few animals (less than three animals), these samples are, where possible, combined with neighbouring years of the same herd. Systematic factors regarded in the multi-trait animal model are the herd-year, sex (male, female), birth type (singleton, multiple), month of birth, calving number, maternal age class (first calving with three age classes, second calving with two age classes, third and fourth calving with one age class, and all further calvings are subsumed in one class) and an age correction to the target age (200 days or 365 days) using linear covariates. The following genetic effects are estimated:

- Random animal effect and EBVs for all traits;
- Maternal genetic effect and maternal EBVs for the 200-day weight.

The total relative breeding value for meat production (RBV-Total) includes EBV-mat-DG 200d, EBV-DG 365d, and EBV-MC 365d, with weights of 40%, 40%, and 20%., respectively. The estimated breeding values and the RBV-Total are published as relative breeding values on a scale with a mean of 100 and a standard deviation of 12 points [18]. In beef, dual-purpose, and dairy cattle, all officially released EBVs are scaled to 100 ± 12 points [18]. De-regressed proofs were calculated using the reliability (r^2^) of the RBV-Total and weighing the deviation of EBVs from the parental average of EBVs by (1/r^2^) [19].

### 2.5. Questionnaire

In addition, a questionnaire was designed to find out how informed the breeders are about progressive ataxia and whether any previous cases of ataxia or animals with ataxia-like signs have been observed. This questionnaire was sent to every breeder who had made use of diagnostic testing in our lab. In cases where a homozygous or heterozygous mutated animal was detected, the farmer who owned it was asked to fill in a further questionnaire before the test result was sent out; this questionnaire sought to determine whether possible clinical signs were observed in these animals in the present and/or past. This information should assist us in checking whether the genetic test is reliable.

### 2.6. Statistical Analysis

All pedigree data were recorded with Opti-Mate, version 4.2 [20]. Calculation of allele and genotype frequencies as well as Chi-square tests for Hardy–Weinberg equilibrium (HWE) were performed using PROC ALLELE of SAS, version 9.4 (Statistical Analysis, Cary, NC, USA, 2023). To determine associations between genotypes, performance traits, and EBVs, the GLM (general linear model) procedure of SAS was employed. The evaluation of the pedigree data for frequently observed sires was performed using Opti-Mate, version 4.2 [20], and SAS.

The model for growth traits included the fixed effects of the *KIF1C:c.608G>A* genotype and sex of the animal for all 1315 animals with genotypes (model 1). Contrasts between effect levels were estimated using the option ‘estimate’ of SAS within the GLM procedure.

An extended model, which included only the progeny of sires that were found to be segregating for the *KIF1C* A-allele, accounted for the sire effect within the *KIF1C* genotype in addition to sex and *KIF1C* genotype for a subset of 287 animals (model 2). Only sires with at least two progeny, each with a homozygous G/G and a heterozygous G/A genotype, were regarded, resulting in 46 sires. A mixed general linear model that considered the sire effect within the *KIF1C* genotype to be randomly distributed gave the same results. A further extension of model 2 was tested by adding a random farm-year effect.

## 3. Results

### 3.1. Genotype Frequencies

A total of 1315 samples were genotyped for the *KIF1C* mutation (c.608G>A). Thereof, 293 animals (22.28%) were heterozygous carriers and eight (0.61%) were homozygous mutated (Table 1).

Of the 324 healthy control samples from different beef cattle breeds, 1 female German Angus and 1 of the 4 examined German Angus bulls were heterozygous *KIF1C* mutants (Table 2).

### 3.2. Allele and Genotype Frequencies

The *KIF1C* mutant allele frequency in Charolais was 0.1175, with an allele count of 309; there were 2630 alleles in total. The comparison between the observed and expected genotype frequencies revealed a significant deviation from HWE. We grouped the animals according to their age at sampling in five age classes. The genotype frequencies within the age classes were in HWE, with the exception of animals > 24 months of age (Table 3).

### 3.3. Comparison of Performance Data and Estimated Breeding Values by Genotypes

Significant differences between homozygous and heterozygous mutated on the one hand and homozygous mutated and wild-type animals on the other hand were observed for birth weight using model 1: EBV-DG 200d and 365d, EBV-MC 200d and 365d, de-regressed RBV-Total and RBV-Total; this was the case even when only three animals were homozygous mutant (Table 4). Birth weight was significantly lower in homozygous A/A animals compared to the G/G and G/A genotypes by 7.9906 kg and 8.1111 kg, respectively; meanwhile, the de-regressed RBV total was significantly higher in homozygous A/A animals by 30.9365 and 30.5243 units, respectively. The greatest differences in EBVs were seen in muscle conformation with contrasts of 14.5406 and 16.0444 points between homozygotes and even with significant values of 0.9569 and 1.6653 between heterozygotes and wild-type animals, respectively. None of the contrasts for WW 200d were significant. For YW 365d, no data were available for homozygous carriers. Means, standard deviations, and ranges of body weight, ages at weighing, and EBVs for the different *KIF1C* genotypes are given in Supplementary Materials Table S3. In addition, contrasts relative to the mean values and the standard deviations of the respective traits are shown for the different *KIF1C* genotypes in Supplementary Materials Table S4.

The data show significant additive effects of the *KIF1C* A allele on birth weight, daily weight gain at 200 and 365 days of age, muscle conformation at 200 and 365 days of age, and the RBV-total (Table 5). In addition, significant negative dominant effects on muscle conformation and daily weight gain are obvious. Birth weight is affected by a positive dominant effect of the *KIF1C* A allele. Maternal genetic effects do not play a role in the daily weight gain of calves.

Model 2, accounting for the sire effect within the *KIF1C* genotype in addition to sex and the *KIF1C* genotype for a subset of 287 animals, provided significant *p*-Values for the birth weight, EBVs for daily weight gain, and scores for muscle development: RBV-Total and de-regressed RBV-Total (Table 6). Herd-year effects were not significant for birth or 200-day weights and were therefore not considered in model 2. Heterozygous animals did not significantly differ from homozygous wild-type animals in any of the traits analysed but birth weight, resulting in significant contrasts.

### 3.4. Pedigree Analysis

Animals under study were sired by 396 bulls, of which 10 (2.5%), 160 (40.4%), and 212 (53.5%) were registered in the Canadian, German, and French herdbooks, respectively. The remaining 14 bulls were from herdbooks other than the Canadian, German, and French ones. In this study, we obtained 6 (1.5%) and 27 (6.8%) samples from German and French bulls, respectively, for genotyping. The Canadian, German, and French bulls sired 27, 489, and 770 progeny, respectively; of these, most progeny had the homozygous wild-type *KIF1C* genotype: 21 (77.8%), 372 (79.0%), and 598 (77.7%), respectively.

Five different bulls sired eight homozygous mutated Charolais progeny (Table 7). There was no information about the sire for one homozygous mutated animal. These four bulls had no common paternal ancestor up to the paternal and maternal grandsire. Except one German bull, all the other three sires had French origin. The most frequently used bull (Bull A) sired 3 homozygous, 17 heterozygous mutant, and 11 homozygous wild-type progeny. This French bull was born in 2013, received a Ib breeding award in 2019, and carries one copy of the mutant *KIF1C* allele. Out of the 17 heterozygous mutant progeny of sire A, 14 received the *KIF1C* A-allele from this bull; in 2 cases, the genotype of the dam was unknown and in one case the dam was heterozygous. A further French bull with an unknown genotype sired 11/19 heterozygous mutant progeny. A heterozygous genotype has to be assumed for sires with homozygous mutated offspring. The genotypes of the dams and sires were determined in this study. At this time, the genotypes of the sires were not publicly available from the breeding organizations. Of the Canadian, German, and French bulls, 6 (6/27 = 22.2%), 116 (116/489 = 23.78%), and 166 (166/770 = 21.6%) heterozygous mutant progeny were sired, respectively. The dams of these progeny were homozygous wild-type in 1 (1/6 = 16.7%), 17 (17/116 = 14.7%), and 43 (43/166 = 37.0%) cases, respectively.

For the two heterozygous mutated German Angus, pedigree data were only available for the male animal. The male individual is a purebred German Angus bull registered in the herdbook, division A, with at least three generations of purebred German Angus.

### 3.5. Evaluation of the Survey

The questionnaire was answered by 35 farmers who took part in our study (Supplementary Materials, Table S5). At this time, they kept a total of 2505 beef cattle, with Charolais cattle accounting for 89.6% of the total. Natural service sires were used for the majority of the cows, accounting for 50.6%. It is important to note that 60% of the breeders had already heard of progressive ataxia in Charolais cattle before we informed them. Of these, 62% were informed by the breeding association or the breeding manager; 14% were informed by their veterinarian. The selection of bulls who were free of the mutant *KIF1C* allele had been reported by 66% of the farmers; it should be noted that 48% of them started doing so after receiving our information letter. One of the farmers participating in the survey had been conducting selections against ataxia since 2010. Of the 35 farmers, 3 had reported that eight animals were showing signs indicative of progressive ataxia. Six of them were confirmed through genetic testing. On six other farms, there were a further twelve individuals who showed signs indicating progressive ataxia according to the report of the farmers. However, these animals had already left the farm before the samples were sent to Hannover for genetic testing.

Of all the farms surveyed, 13 (37%) had already seen animals with weakness in the hind limbs, followed by 31% who reported that they observed incidents of uncoordinated gait. Furthermore, permanent recumbency (14%), crossed limbs (11%), irregular heel of urine (9%), and abrupt head movements (3%) were noted. Confirmation of progressive ataxia was not possible in these cases because these observations were made before genetic testing was available.

Of all the participating farms, 83% reported that they would like to have their own sires genetically tested in the future. And 80% reported that they would also want to test all their female breeding cattle. The farmers felt there would be no need for genetic testing among fattening animals (0%) because clinical signs are unlikely at that age and the animals are not used for breeding.

With the exception of two breeders, all participants reported that they consider it necessary to reduce the frequency of the mutated allele through preventing heterozygous animals from breeding. Two farmers were not in support of excluding all heterozygous animals from breeding because heterozygous female animals can be used for mating with homozygous-free bulls. These two participants are among the 97% of farmers who were requesting the publication of the *KIF1C* genotypes of all artificial insemination bulls. Most of the farmers agreed that *KIF1C* genotypes should be made public for auction animals (83%) and bulls at licensing (89%).

The questionnaire revealed that 6/8 homozygous mutated animals showed clinical signs before or concurrently with sampling. The two animals without clinical signs were 13 and 64 days old at the time of sampling (Table 8).

## 4. Discussion

The questionnaire used in this study showed that progressive ataxia was not yet known to all participating Charolais breeders, despite the widespread distribution of the disease, its long persistence, and its impact on animal welfare. This shows how important it is to inform breeders, breeding associations, and veterinarians of new developments in the elucidation of inherited defects and opportunities of genetic testing. The survey was also useful for breeders in informing their future breeding decisions; additionally, it supported them in recognizing the extent to which the mutated *KIF1C* genotype may impact their breeding animals and the broader Charolais population.

The present study showed that the *KIF1C* mutant allele segregated with a similar frequency in the German Charolais population as in the French population [10]. The reason for this result may be explained by the heavy use of French Charolais bulls in Germany and the close relationships of bulls registered in the German herdbook with the French population. There were some popular Charolais bulls that had increased the *KIF1C* mutant allele frequency beyond the expected proportion, such as bull A. Due to the unknown etiology of this condition and the persistence of this defect over many decades, the mutated *KIF1C* allele was imported from the French population via females and males. An introgression into the Uckermärker population through French Charolais bulls resulted in a similar frequency of the mutated *KIF1C* allele as that in the German Charolais population [9].

The genotypic distribution across all samples did not reveal HWE. This may be due to the fact that affected animals have already been culled by breeders or that homozygous animals may have higher embryonic mortality or lower survival rates after birth. Evaluation by age group showed that the genotypes from animals under 24 months are in HWE. Thus, we propose that animals with increasingly worsening signs of ataxia should leave the farm, even if genetic testing has not yet been performed. With this genetic test, farmers could test animals indicating signs of illness and then decide how to proceed with these animals.

The presence of two heterozygous mutated German Angus cattle may indicate that there was also an introgression of Charolais in the 1950s–1970s when German Angus was created through crossbreeding of Aberdeen Angus bulls with different cattle breeds in Germany. However, it was not possible to prove this assumption because the depth of pedigree data was insufficient. The heterozygous German Angus bull was born in 2004, registered in the herdbook as a purebred animal, and mostly used for crossbreeding with dairy cows. We could not follow-up on the progeny of this bull; thus, we could not evaluate the dissemination of the *KIF1C* A allele in its progeny. We assume from the literature [10] and our data that the *KIF1C* A allele is not present in dairy breeds; therefore, it is very unlikely that cases of progressive ataxia may be found in the progeny of this German Angus bull. Cases of ataxia in Angus cattle were described for calves aged between 4 days and 3 months of age [21] and for an 18-month-old Angus cow [22]. Considering the age of 18 months in this case report, a reference to ataxia in Charolais cattle is permissible. However, no myelin loss was found in the brain or spinal cord [22]. In a further case with ataxia and urinary incontinence in Angus cattle, a sorghum poisoning was issued [23]. A survey of the Angus breeders and further studies with larger sample sizes can clarify if this mutation segregates in the German Angus population and whether it triggers the same signs at the same age. Six of eight Charolais cattle tested for the homozygous mutant have also shown clinical signs. The two homozygous mutated animals without any clinical findings were 13 and 64 days old at the time of sampling. Clinical findings are very unlikely at this age. Thus, we can confirm the validity of the genetic tests based on clinical observations.

The association between growth traits and the mutated *KIF1C* A allele could be confirmed. Duchesne et al. [10] found significant contrasts between homozygous wild-type and heterozygous mutated genotypes for muscular development at 7 months, weight at 7 and 24 months, and skeletal development at 30 months. Effects were largest in 30 months-old animals. In the present study, we employed EBVs and de-regressed EBVs to correct for non-genetic effects; with the BLUP animal model, all contributions found through the relationship matrix were used to estimate the additive genetic values (EBVs) of the animals. In agreement with the French study, we found significant differences between heterozygous and wild-type homozygous animals for EBV-MC 200d and EBV-MC 365d, but no differences were found in any EBVs for daily weight gain. The muscle conformation scores are comparable to muscle development at 7 months in the French report [10]. In the present study, relative differences were lower for EBV-MC 200d and EBV-MC 365d with values of 1.58% and 0.92%, respectively (contrast per mean as percentage); alternatively, they were found to be similar for the contrasts per standard deviation, with values of 0.22 and 0.14, respectively, compared to the French data, with values of 2.5% and 0.17, respectively [10]. However, contrasts for growth traits between homozygous A/A and wild-type G/G animals were not significant in the French study [10]. In the present study, the relative differences for the de-regressed RBV-Total between A/A and G/A as well as G/G animals were at 27.80–28.18% (contrasts per mean as percentage) and 1.57–1.59 (contrasts per standard deviation). We found significant additive and dominant effects on birth weight, daily weight gain, and muscle conformation, but not on maternal genetic effects. Allelic substitution effects were not significant because we did not find differences between heterozygous and homozygous wild-type animals in growth traits. Further data on the performances of homozygous mutant animals would be necessary to corroborate the estimates presented here, because only three homozygous mutated animals had performance data. An extended model for the sire effects within the *KIF1C* genotype resulted in very similar results. This may be indicative for a pleiotropic *KIF1C* effect, even if only a small subset of the data could be employed for this analysis. Nevertheless, the gene action of the *KIF1C* A allele may be assumed to be more complex than previously reported [10]. Further research using genotype data from single nucleotide polymorphism arrays may be used to distinguish between possible pleiotropic and linkage disequilibrium effects on growth parameters or even other traits [24,25,26,27]. Due to linkage disequilibrium, alleles from the loci in close proximity to *KIF1C* on BTA19 are more likely to be transmitted together to the next generation. In addition, assortative mating, drift, and migration can lead to the joint transmission of separate loci at different sites on other chromosomes [24]. Pleiotropy of a gene variant causes associations between different traits because the encoded protein has more than one target or influences several pathways or a pathway with several downstream effects [24,25,26,27]. Assuming pleiotropy for the *KIF1C* genotype on growth traits, breeders may be constrained in improving the daily weight gain and muscular development values through selection for the *KIF1C* A-allele. Selection for the *KIF1C* A allele must be banned for the welfare of homozygous mutant animals and is therefore not an option for breeders. This study confirmed the small advantages of heterozygous mutated animals in muscle development at the ages of 200 and 365 days in model 1 only [10]; this was not found to be the case in model 2, which accounted for sire effects. However, much larger effects were found for homozygous mutated animals and, thus, for additive effects. Possibly, the late onset of this condition in homozygous mutant animals may have driven the persistence of the defective *KIF1C* A allele in male and female breeding animals in a heterozygous state and not the superiority of heterozygous mutated animals. In this way, the superiority of homozygous mutant animals in daily weight and muscle development could be exploited without the risk of losing breeding animals. Further variants may influence the progressing of clinical signs; there might have been selection for late onset of the progressive ataxia in Charolais.

The limitations of our study were that the subset to be tested for a possible pleiotropic effect of the *KIF1C* variant was quite small; thus, a rigorous study employing SNP array data could not be performed. Therefore, further research is needed to clarify whether loci that are in close linkage disequilibrium with *KIF1C* contribute to the association with growth traits found in this study. Searching for QTL and loci associated with growth traits in the animal genome database (https://www.animalgenome.org/cgi-bin/QTLdb/BT, accessed on 20 November 2023) found four growth-associated loci at 23.9–26.9 Mb on BTA19 (cow reference assembly ARS UCD1.2, *KIF1C* located at 26,390,112–26,412,206 bp on BTA19) and two QTL (26.2 and 28.1 Mb on BTA19). Therefore, the possibility that loci are in close linkage disequilibrium with *KIF1C* cannot be ruled out on the basis of our results.

The prevalence and the relationship between progressive ataxia and performance for meat production highlights the need for comprehensive genetic testing and publicly available genotypes.

## 5. Conclusions

The high demand for a genetic test shows that progressive ataxia is an important issue for Charolais breeders. The high frequency of the *KIF1C:*c.608G>A variant in the German Charolais population underlines the importance of genetic testing to avoid welfare problems in yearlings and older animals. The further spread of this lethal variant in the Charolais population can be reduced if beef cattle breeders are aware of this lethal condition and informed about how to prevent it and eliminate the defective allele in the long term. Estimates of additive and dominant effects provided further insights into how the mutant *KIF1C* A variant affects growth parameters in Charolais. The persistence of the defective *KIF1C* A variant through breeding of heterozygous cows and bulls may be driven through the superiority of homozygous A/A animals in muscling and weight gains. Further research may clarify whether pleiotropic effects or linkage disequilibrium might play a role.

## Figures and Tables

**Table 1:** Genotype counts and frequencies of the *KIF1C:c.608G>A* genotypes in 1315 German Charolais cattle.

Genotype	Male	Female	Total	Frequency (%)
G/G	164	850	1014	77.11
G/A	41	252	293	22.28
A/A	1	7	8	0.61

**Table 2:** Genotyping of 324 animals from eight different cattle breeds for the *KIF1C* mutation.

Breed	Number of Animals		
	Wild-Type G/G	Homozygous A/A	Heterozygous G/A
German Angus	95	0	2
Blonde d’Aquitaine	12	0	0
German Brown	11	0	0
German Fleckvieh	98	0	0
German Holstein	11	0	0
Limousin	11	0	0
Salers	11	0	0
Vorderwälder	73	0	0

**Table 3:** Tests for Hardy–Weinberg equilibrium using chi-square tests and allele frequencies with their standard errors (SEs) for all samples and by age groups. *p*-Values indicate whether the samples significantly (*p*-Value < 0.05) deviate from Hardy–Weinberg equilibrium.

Samples	n	Chi-Square	*p*-Value	Allele Frequency and SE		
				A	G	SE
All Samples	1315	7. 2905	0.0069	0.1175	0.8825	0.006
Age groups						
0–6 months	146	0.0282	0.8667	0.1233	0.8767	0.019
7–12 months	229	0.4744	0.4910	0.1157	0.8843	0.015
13–18 months	88	0.7639	0.3821	0.0852	0.9148	0.020
19–24 months	95	0.0027	0.9589	0.1474	0.8526	0.026
>24 months	757	8.6338	0.0033	0.1169	0.8831	0.008

**Table 4:** Comparisons of performance data and estimated breeding values for daily weight gain (EBV-DG) and score for muscle development (EBV-MC) at day 200 and 365 with their *p*-Values using model 1.

Trait	A/A-G/G		A/A-G/A		G/A-G/G	
	Contrast	*p*-Value	Contrast	*p*-Value	Contrast	*p*-Value
Birth weight (kg)	−7.9906	0.0111	−8.1111	0.0104	0.1204	0.7779
WW 200d (kg)	13.6574	0.7559	10.9044	0.8052	2.7530	0.6678
YW 365d (kg)		-		-	7.6448	0.2632
EBV-DG 200d	12.7433	0.0042	12.2102	0.0063	0.5331	0.3482
EBV-DG 365d	9.3397	0.0294	8.9626	0.0375	0.3771	0.4914
EBV-MC 200d	16.0444	0.0030	14.3791	0.0011	1.6653	0.0030
EBV-MC 365d	14.5406	0.0001	13.5837	0.0004	0.9569	0.0477
RBV-Total	12.8589	0.0046	12.6023	0.0058	0.2566	0.6584
De-regressed RBV-Total	30.9365	0.0051	30.5243	0.0060	0.41215	0.7703
EBV-mat-DG 200d	0.0940	0.9767	0.62505	0.8470	−0.53102	0.1983

EBV = estimated breeding value on a scale of 100 ± 12 points; EBV-mat = estimated maternal breeding value; MC = score for muscle development; WW = weaning weight; YW = yearling weight; DG = daily weight gain; RBV-Total = relative estimated total breeding for meat production; significance threshold for *p*-Values set at <0.05.

**Table 5:** Half of the difference between homozygous (contrast between homozygous, corresponding to the additive effect) and the difference between heterozygous and the average of the homozygous (contrast between heterozygous and the mean of homozygous, corresponding to the dominant effect) with their *p*-Values for performance data and estimated breeding values for daily weight gain (EBV-DG) and score for muscle development (EBV-MC) at day 200 and 365 using model 1.

Trait	Contrast between Homozygous		Contrast between Heterozygous and the Mean of Homozygous	
	Estimate ± SE	*p*-Value	Estimate ± SE	*p*-Value
Birth weight (kg)	−3.9953 ± 1.5713	0.0111	4.1157 ± 1.6168	0.0111
WW 200d (kg)	6.8287 ± 21.9627	0.7559	−4.0757 ± 22.7023	0.8576
YW 365d (kg)	n.e.		n.e.	
EBV-DG 200d	6.3716 ± 2.2194	0.0042	−5.8385 ± 2.2766	0.0105
EBV-DG 365d	4.6698 ± 2.1412	0.0294	−4.2927 ± 2.1964	0.0509
EBV-MC 200d	8.0222 ± 2.1894	0.0003	−6.3569 ± 2.2459	0.0047
EBV-MC 365d	*7*.2703 ± 1.8860	0.0001	−6.3134 ± 1.9347	0.0011
RBV-Total	6.4294 ± 2.2668	0.0046	−6.1729 ± 2.3252	0.0080
De-regressed RBV-Total	15.4682 ± 5.5146	0.0051	−15.0561 ± 5.6567	0.0079
EBV-mat-DG 200d	0.0470 ± 1.6120	0.9767	−0.5780 ± 1.6535	0.7267

EBV = estimated breeding value on a scale of 100 ± 12 points; EBV-mat = estimated maternal breeding value; MC = score for muscle development; WW = weaning weight; YW = yearling weight; DG = daily weight gain; RBV-Total = relative estimated total breeding for meat production; n.e. = not estimable; significance threshold for *p*-Values set at < 0.05.

**Table 6:** Half of the difference between homozygous (contrast between homozygous, corresponding to the additive effect) and difference between heterozygous and the average of the homozygous (contrast between heterozygous and the mean of homozygous, corresponding to the dominant effect) with their *p*-Values for performance data and estimated breeding values for daily weight gain (EBV-DG) and score for muscle development (EBV-MC) at day 200 and 365 using model 2.

Trait	Contrast between Homozygous		Contrast between Heterozygous and the Mean of Homozygous	
	Estimate ± SE	*p*-Value	Estimate ± SE	*p*-Value
Birth weight (kg)	−3.8043 ± 1.4391	0.0089	3.0915 ± 1.5670	0.0499
WW 200d (kg)	−3.9582 ± 18.3862	0.8298	−5.7615 ± 20.1427	0.7752
YW 365d (kg)	n.e.		n.e.	
EBV-DG 200d	5.7247 ± 1.5731	0.0003	−5.3524± 1.6998	0.0019
EBV-DG 365d	4.0262 ± 1.4030	0.0045	−3.7021 ± 1.5160	0.0154
EBV-MC 200d	6.8759 ± 1.5320	0.0001	−6.5486 ± 1.6553	0.0001
EBV-MC 365d	6.1155 ± 1.1602	0.0001	−5.9908 ± 1.2536	0.0001
RBV-Total	5.5508 ± 1.6127	0.0007	−5.9437 ± 1.7426	0.0008
De-regressed RBV-Total	13.1740 ± 3.6824	0.0004	−15.2366 ± 3.9790	0.0002
EBV-mat-DG 200d	0.1311 ± 1.2616	0.9173	−0.9014 ± 1.3632	0.5092

EBV = estimated breeding value on a scale of 100 ± 12 points; EBV-mat = estimated maternal breeding value; MC = score for muscle development; WW = weaning weight; YW = yearling weight; DG = daily weight gain; RBV-Total = relative estimated total breeding for meat production; n.e. = not estimable; significance threshold for *p*-Values set at <0.05.

**Table 7:** Most frequently observed sires by the genotypes of their progeny (progeny genotype) with the total number of progeny tested (N total), number of homozygous or heterozygous mutant progeny (N per genotype), wild-type *KIF1C* genotype of the dams (G/G), and the sire’s *KIF1C* genotype. For animals homozygous for the *KIF1C* A allele, all sires are given. For heterozygous carriers, the top ten most common sires are shown.

Progeny		Dam		Sire		
Genotype	N Total	N per Genotype	G/G	Identification	Genotype	Origin ^1^
A/A	31	3	0	A	G/A	FR
	2	2	0	B	unknown	FR
	20	1	0	C	unknown	FR
	18	1	0	D	unknown	DE
	-	1	0	unknown	unknown	unknown
G/A	31	17	14	A	G/A	FR
	19	11	3	E	unknown	FR
	19	7	0	F	unknown	DE
	13	6	2	B	unknown	FR
	12	6	1	G	G/A	DE
	9	6	4	H	unknown	FR
	23	5	1	I	unknown	FR
	8	5	2	J	G/A	FR

^1^ DE = Germany; FR = France.

**Table 8:** Survey on the eight homozygous mutated animals with their age at sampling and status of clinical signs before sampling and concurrently at date of sampling.

Animal	Age (Days)	Current Clinical Signs	Clinical Signs before Testing
1	642	Yes	Yes
2	333	Yes	No
3	325	Yes	No
4	731	Yes	Yes
5	744	Yes	Yes
6	759	Yes	Yes
7	13	No	No
8	64	No	No

## Data Availability

Restrictions apply to the availability of these data. Data were obtained from the participating Charolais farms and are available from the authors at a reasonable request and with the permission of the Charolais breeders.

## Supplementary Materials

The following supporting information can be downloaded at: https://www.mdpi.com/article/10.3390/ani14030366/s1: Figure S1. Genotyping using a PCR-RFLP for *KIF1C*:g.27041449G>A for animals 1 to 18. Table S1. Announcement of genetic testing for progressive ataxia in Charolais cattle at the Institute for Animal Breeding and Genetics, University of Veterinary Medicine Hannover (Foundation). Table S2. Primer sequences used for validation of the *KIF1C*:g.27041449G>A mutation using a restriction fragment length polymorphism (RFLP). Table S3. Body weights in kg, age in days at weighing and relative estimated breeding values (EBV) by genotype for both sexes. Table S4. Comparisons of performance data and estimated breeding values for daily weight gain (EBV-DG) and scores for muscle development (EBV-MC) at day 200 and 365 expressed as percentages per mean or relative per standard deviation (model 1). Table S5. Evaluation of the survey among beef cattle breeders.
