# Supplementary material for: Genotyping KIF1C (c.608G>A) Mutant Reveals a Wide Distribution of Progressive Ataxia in German Charolais Cattle
Source: Animals (Basel). 2024 Jan 23;14(3):366. doi: 10.3390/ani14030366 (PMC10854487; doi:10.3390/ani14030366)
Supplement: Supplementary file 1 [file animals-14-00366-s001.zip › animals-2784564-supplementary.pdf]

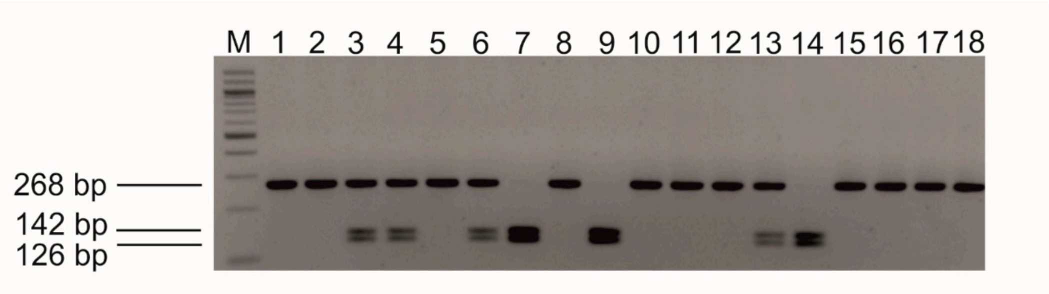

Figure S1. Genotyping using a PCR-RFLP for *KIF1C*:g.27041449G>A for animals 1 to 18. The lane M is a 100-bp ladder marker. All the homozygous mutant animals, which were found in this study with their parents, if available, can be seen in this gel picture. Two families (first: 1-6, second: 10-13), homozygous mutant animals without typed parents (8, 15-18) and three wild types (7, 9, 14) were genotyped in this picture. The wild type alleles had 126 and 142 base pair fragments (G/G), the homozygous mutant with a fragment length of 268 base pairs (A/A) and the heterozygous with all of these alleles (G/A). Animals 1 and 2 were full siblings, animals 3 and 4 their parents and animal 5 was a paternal half sibling. Animals 10 and 11 were full siblings, animal 12 a maternal half sibling and animal 13 was the dam.

Table S1. Announcement of genetic testing for progressive ataxia in Charolais cattle at the Institute for Animal Breeding and Genetics, University of Veterinary Medicine Hannover (Foundation).

Institut für Tierzucht und Vererbungsforschung
Stiftung Tierärztliche Hochschule Hannover

Progressive Ataxie beim Charolais Rind

Die progressive Ataxie ist ein tödlich verlaufender Erbfehler (Letalmutante) infolge von irreversiblen Veränderungen in Gehirn und Rückenmark. Die peripheren Nerven weisen keine Veränderungen auf. Dieser Erbfehler ist heimtückisch, da bei Kälbern keine Anzeichen dieser Erkrankung zu erkennen sind. Die Erkrankung beginnt i.d.R. schleichend im Alter von 18 bis 24 Monaten mit Schwäche in den Hinterbeinen und Überkreuzen der Beine. Bei Absetzern sind Anzeichen dieser Erkrankung nur äußerst selten zu erkennen. Über 2 Jahre alte Tiere können ebenfalls noch erkranken. Der Auslöser der progressiven Ataxie ist eine einzige genetische Veränderung (Mutation) in dem Gen *KIF1C*. Dieses Gen ist bei Tieren mit dem Erbfehler progressive Ataxie funktionslos. Infolgedessen setzt in spezifischen Gehirn- und Rückenmarksarealen ein zunehmender Abbau von weißer Substanz ein, woraus die zentralnervösen Ausfallerscheinungen mit unkoordiniertem Gang, sich verschlimmernden Bewegungsstörungen und letztendlich Festliegen resultieren. Bei einigen Tieren können abrupte Kopfbewegungen bei Aufregung sowie stoßweiser Harnabsatz beobachtet werden. Männliche und weibliche Tiere sind gleichermaßen betroffen.

Für den Erbfehler progressive Ataxie wurde ein Gentest in Frankreich entwickelt, mit dem bereits beim Kalb eine zweifelsfreie Diagnose möglich ist und die Anpaarungen so durchgeführt werden können, dass keine Erbfehlerträger mehr auftreten können. Diese Mutation in dem *KIF1C* Gen wird nach Mendel autosomal-rezessiv vererbt. Das bedeutet, dass auf beiden Chromosomen das mutierte *KIF1C* Gen vorhanden sein muss, damit das Rind die progressive Ataxie ausprägt. Nur wenn beide Eltern diese Mutation in ihrem Erbgut tragen, kann es Nachkommen mit diesem Erbfehler geben. Besonders auf der Hut müssen die Charolais Züchter und Halter sein, wenn sie Jungbullen zum Decken einsetzen. Diese Jungbullen können unerkannt Erbfehlerträger sein, da die ersten Anzeichen i.d.R. erst im Alter von 18-24 Monaten sichtbar werden. Für das Sperma von KB-Bullen und Deckbullen müssen Züchter und Tierhalter sicherstellen, dass die Tiere auf das defekte *KIF1C* Gen getestet und anlagefrei für die *KIF1C* Mutation sind. Ist das nicht mehr möglich, so sollten die Nachkommen getestet werden, um ein Auftreten von progressiver Ataxie zu einem späteren Zeitpunkt im Leben auszuschließen.

Charolaisrinder mit dem mutierten *KIF1C* Gen auf einem Chromosom (Anlageträger) erkranken nicht an progressiver Ataxie, können jedoch das defekte Gen mit einer Wahrscheinlichkeit von 50% an ihre Nachkommen

weitergeben. Diese Rinder zeigen höhere Zuwachsraten, weshalb sehr wahrscheinlich eine Selektion auf diese Ausprägungsform des *KIF1C* Gens stattfand. Das erklärt die lange währende Persistenz dieser Mutation und die aktuell hohe Frequenz dieser Mutation von 13% in der französischen Charolais Population. Anlageträger dürfen nicht miteinander verpaart werden, da hier das Risiko besteht, mit einer Wahrscheinlichkeit von 25% Erbfehlerträger zu erhalten.

Züchter und Halter von Charolais oder Kreuzungstieren mit Charolais können am Institut für Tierzucht und Vererbungsforschung der Stiftung Tierärztliche Hochschule Hannover den DNA Test für progressive Ataxie in Auftrag geben. Der DNA Test wird entsprechend der Originalpublikation von Duchesne et al. 2018 (Progressive ataxia of Charolais cattle highlights a role of *KIF1C* in sustainable myelination, PLoS Genet 14(8): e1007550) durchgeführt. Für die Anforderung des DNA Tests soll das beiliegende Formblatt verwendet werden. Das Formblatt muss unterschrieben sein und kann per Email, Fax oder Post zugesandt werden. Das Probenmaterial können Haarwurzeln (ca. 50) aus dem Schwanz oder eine EDTA-Blutprobe (3-5 ml) sein. Eine Anleitung zur Probenahme und zum Versand ist ebenfalls hier zu finden. **Bis zum 30. Juni 2019 wird der Test für progressive Ataxie kostenlos angeboten.**

Das Testergebnis für die Ausprägung des *KIF1C* Gens wird für jedes einzelne Tier dem Einsender wie folgt mitgeteilt:
Anlagefrei (homozygot *KIF1C* G/G)
Anlageträger (heterozygot *KIF1C* A/G)
Merkmalsträger (homozygot *KIF1C* A/A)

Sollten Tiere Anzeichen von Ataxie aufweisen und das Testergebnis anlagefrei oder Anlageträger sein, so sollten Sie sich mit uns in Kontakt setzen. In diesem Fall sollte das Tier nicht sofort verwertet werden, damit noch weitergehende Untersuchungen möglich sind. Alle Informationen werden von uns streng vertraulich behandelt und nicht an Dritte weitergegeben.

Kontaktdaten:

Prof. Dr. Ottmar Distl
Institut für Tierzucht und Vererbungsforschung
der Stiftung Tierärztliche Hochschule Hannover
Bünteweg 17 p
30559 Hannover
Fax: 0511-953-8582
E-Mail: ABGLab@tiho-hannover.de oder ottmar.distl@tiho-hannover.de
Telefon: 0511-953-8875

Table S2. Primer sequences used for validation of the *KIF1C:*g.27041449G>A mutation using a restriction fragment length polymorphism (RFLP). Location of *KIF1C* on the assembly ARS-UCD2.0, primer pairs, amplicon size (AS) in base pairs (bp), annealing temperature (AT), restriction enzyme and incubation temperature (IT) are given.

**Gene**	*KIF1C*
**Location**	BTA19, NC_037346.1 (26390125-26412273), ARS-UCD2.0
**Polymorphism**	g.27041449G>A
**Forward primer (5′-3′)**	GCTGGTCTCATTCATGTTGGT
**Reverse primer (5′-3′)**	AGACCTTTTGAACCCCAAGAG
**AS (bp)**	268
**AT (°C)**	59
**Restriction enzyme**	BstUI
**IT (°C)**	60

Table S3. Body weights in kg, age in days (d) at weighing and estimated breeding values (EBV) by genotype for both sexes.

Genotype	Trait [1]	n	Mean	Standard deviation	Median	Range
A/A	Birth weight (kg)	3	36.0	1.0	36.0	35.0-37.0
	Age 200d (days)	3	184.6	7.5	189.0	176.0-189.0
	WW 200d (kg)	3	296.3	34.0	287.0	268.0-334.0
	Age 365d(days)	0	-	-	-	-
	YW 365d (kg)	0	-	-	-	-
	EBV-DG 200d	3	115.0	7.0	112.0	110.0-123.0
	EBV-DG 365d	3	110.6	6.4	108.0	106.0-118.0
	EBV-MC 200d	3	120.3	3.2	119.0	118.0-124.0
	EBV-MC 365d	3	118.0	3.5	116.0	116.0-122.0
	RBV-Total	3	116.0	6.1	113.0	112.0-123.0
G/A	Birth weight (kg)	203	44.6	6.0	45.0	25.0-65.0
	Age 200d (days)	175	192.6	49.8	189.0	91.0-280.0
	WW 200d (kg)	175	290.0	76.2	296.0	122.0-502.0
	Age 365d (days)	158	349.8	45.3	345.5	282.0-500.0
	YW 365d (kg)	158	451.8	81.2	437.0	294.0-752.0
	EBV-DG 200d	229	103.3	7.3	104.0	85.0-126.0
	EBV-DG 365d	229	102.4	7.1	103.0	83.0-122.0
	EBV-MC 200d	229	106.4	7.8	107.0	86.0-138.0
	EBV-MC 365d	229	105.0	6.6	104.0	87.0-121.0
	RBV-Total	229	104.0	7.9	105.0	78.0-128.0
G/G	Birth weight (kg)	801	44.5	5.5	44.0	19.0-72.0
	Age 200d (days)	701	195.4	50.4	197.0	91.0-280.0
	WW 200d (kg)	701	286.9	76.5	286.0	94.0-529.0
	Age 365d (days)	584	351.8	50.6	344.0	281.0-500.0
	YW 365d (kg)	584	447.7	94.7	431.5	241.0-904.0
	EBV-DG 200d	899	102.8	7.9	103.0	73.0-132.0
	EBV-DG 365d	899	102.0	7.7	102.0	74.0-133.0
	EBV-MC 200d	899	104.7	7.6	105.0	74.0-123.0
	EBV-MC 365d	899	104.0	6.6	104.0	75.0-125.0
	RBV-Total	899	103.8	8.0	104.0	73.0-131.0
All	Birth weight (kg)	1007	44.5	5.6	44.0	19.0- 72.0
	Age 200d (days)	879	194.8	50.2	194.0	91.0-280.0
	WW 200d (kg)	879	287.5	76.3	288.0	94.0-529.0
	Age 365d (days)	742	351.4	49.5	345.0	281.0-500.0
	YW 365d (kg)	742	448.6	91.9	433.0	241.0-904.0
	EBV-DG 200d	1131	102.9	7.8	103.0	73.0-132.0
	EBV-DG 365d	1131	102.1	7.6	102.0	74.0-133.0
	EBV-MC 200d	1131	105.1	7.7	105.0	74.0-138.0
	EBV-MC 365d	1131	104.2	6.7	104.0	75.0-125.0
	RBV-Total	1131	103.9	8.0	104.0	73.0-131.0

[1]WW 200d=Weaning weight at an age of 200 days; YW 365d=Yearling weight at an age of 365 days; Estimated breeding values on a scale of 100 ± 12 points; EBV-DG = Estimated breeding value for daily weight gain; EBV-MC = Estimated breeding value for muscle development; RBV-Total = relative estimated total breeding value for meat production.

Table S4. Comparisons of performance data and estimated breeding values for daily weight gain (EBV-DG) and scores for muscle development (EBV-MC) at day 200 and 365 expressed as percentages per mean or relative per standard deviation (model 1).

Trait	(A/A - G/G)/mean	(A/A - G/A)/mean	(G/A - G/G)/mean
Birth weight (kg)	-17.95%	-18.22%	0.27%
WW 200d (kg)	4.75%	3.79%	0.96%
YW 365d (kg)	-	-	1.70%
EBV-DG 200d	12.38%	11.86%	0.52%
EBV-DG 365d	9.15%	8.78%	0.37%
EBV-MC 200d	15.27%	13.68%	1.58%
EBV-MC 365d	13.95%	13.03%	0.92%
RBV-Total	12.38%	12.13%	0.25%
De-regressed RBV-Total	28.18%	27.80%	0.38%
EBV-mat-DG 200d	0.09%	0.62%	-0.53%
**Trait**	**(A/A - G/G)/SD**	**(A/A - G/A)/SD**	**(G/A - G/G)/SD**
Birth weight (kg)	-1.43	-1.45	0.02
WW 200d (kg)	0.18	0.14	0.04
YW 365d (kg)	-	-	0.08
EBV-DG 200d	1.64	1.57	0.07
EBV-DG 365d	1.24	1.19	0.05
EBV-MC 200d	2.09	1.87	0.22
EBV-MC 365d	2.18	2.04	0.14
RBV-Total	1.61	1.58	0.03
De-regressed RBV-Total	1.59	1.57	0.02
EBV-mat-DG 200d	0.02	0.11	-0.10

EBV=Estimated breeding value on a scale of 100 ± 12 points; EBV-mat=Estimated maternal breeding value; MC: score for muscle development; WW= weaning weight; YW=yearling weight; DG: daily weight gain; RBV-Total=relative estimated total breeding for meat production; SD=standard deviation.

Table S5. Evaluation of the survey among beef cattle breeders.

1. Did you already know the genetic defect progressive ataxia in charolais cattle before our information? (SC)

Yes	n=21	60%	No	n=14	40%

1.1 If yes, how were you informed? (SC)

Veterinarian	n=3	14%	Breedingassociation	n=13	62%	Press	n=2	10%	Others	n=3	14%

2. Do you already select bulls for mating which are not carrying the hereditary defect? (SC)

Yes	n=23	66%	No	n=12	34%

2.2 If yes, how long have you been doing this? (SC)

Since our information	n=11	48%	earlier	n=12	52%

3. Were any affected animals diagnosed in your herd before sampling? (SC)

Yes	n=3	9%	No	n=26	74%	Only suspected	n=6	17%

3.1 If yes, how were they diagnosed? (SC)

Veterinarian	n=2	67%	Veterinary government	n=0	0%	Genetic testing	n=1	33%	Others	n=0	0%

4. Have you already had animals with the following symptoms? (MC)

Hind limb weakness	n=13	37%	Uncoordinated gait	n=11	31%	Crossing hind limbs	n=4	11%
Abrupt head movements	n=1	3%	Irregular heel of urine	n=3	9%	Permanent recumbency	n=5	14%

5. Which animals of your herd would you like to examine for progressive ataxia using genetic testing in the future? (MC)

none	n=1	3%	Natural insemination bulls	n=29	83%	Female breeding cattle	n=28	80%	Fattening animals	n=0	0%

6. Is it necessary for you to select animals not carrying the mutated allele? (SC)

Yes	n=33	94%	No	n=2	6%

6.1 If yes in which group of animals? (MC)

Natural insemination bulls	n=30	91%	Artificial insemination bulls	n=31	94%	Female breeding cattle	n=23	70%
Weaned calfs for sale	n=9	27%	Bulls for grading	n=23	70%			

6.2 If no, why not? (SC)

costs	n=0	0%	No need	n=1	50%	others	n=1	50%

7. For which group of animals would you prefer to have the genotype for progressive ataxia? (MC)

Insemination Bulls	n=34	97%	Licensed Bulls	n=31	89%	Auction animals	n=29	83%	Others	n=5	14%

In total, 35 breeders who kept 2505 cattle answered the questionnaire. The question form is indicated for each question with single choice (SC) or multiple choice (MC). For follow-up questions, 100% corresponds to the respective number of participants who selected the corresponding answer option.
